# Refractory Recurrent Pericarditis After Pericardiectomy in a Young Woman

**DOI:** 10.1016/j.jaccas.2021.10.006

**Published:** 2021-12-01

**Authors:** Hassan Mehmood Lak, Chris M. Anthony, Muhammad M. Furqan, Beni Rai Verma, Mohamed Gad, Sanchit Chawla, Farah Yasmin, Deborah H. Kwon, Douglas R. Johnston, Allan L. Klein

**Affiliations:** a Department of Internal Medicine, Cleveland Clinic, Cleveland, Ohio, USA; b Center for the Diagnosis and Treatment of Pericardial Diseases, Section of Cardiovascular Imaging, Robert and Suzanne Tomsich Department of Cardiovascular Medicine, Sydell and Arnold Miller Family Heart, Vascular, and Thoracic Institute, Cleveland Clinic, Cleveland, Ohio, USA; c Dow Medical College, Karachi, Pakistan; d Department of Thoracic and Cardiovascular Surgery, Sydell and Arnold Miller Family Heart, Vascular, and Thoracic Institute, Cleveland Clinic, Cleveland, Ohio, USA

**Keywords:** adhesions, cardiac magnetic resonance imaging, pericardiectomy, recurrent pericarditis, CMR, cardiac magnetic resonance imaging, CRP, C-reactive protein, DHE, delayed hyperenhancement, ESR, erythrocyte sedimentation rate, PPS, postpericardiotomy syndrome, PSIR, phase-sensitive inversion recovery, STIR, short T1 inversion recovery

## Abstract

Pericardiectomy is the recommended treatment for patients with recurrent pericarditis and refractory symptoms despite optimal anti-inflammatory therapy. We present a case of a 40-year-old woman who underwent total pericardiectomy after multiple episodes of pericarditis that was refractory to optimal guideline-derived medical therapy, including anti-inflammatory and biologic agents, who continued to have relapsing symptoms even after pericardiectomy. (**Level of Difficulty: Intermediate.**)

## Central Illustration

**Figure:**
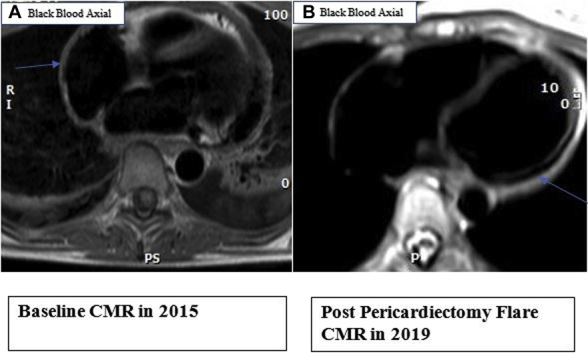

## History of Presentation

A 40-year-old woman presented to the pericardial center with recurrent symptoms of pericarditis. Her symptoms were typical for flares of recurrent pericarditis, which included pleuritic chest pain radiating to her left shoulder, shortness of breath, and constitutional symptoms of feeling unwell and lethargic.

**Learning Objectives**

• To understand the role of serial cardiac magnetic resonance imaging in the treatment of patients with complex recurrent pericarditis.
• To understand the role of pericardiectomy in patients with refractory recurrent pericarditis without the presence of physiologic constriction.
• To be able to understand that a small subset of patients may experience recurrent flares of pericarditis after pericardiectomy and may require novel immunosuppressive therapies and cardiac magnetic resonance imaging for optimal management.

## Medical History

In 2015, the patient underwent mitral valve repair for mitral valve prolapse. Six weeks after cardiac surgery, she experienced a fever of 102 °F, sharp pleuritic chest pain that worsened on lying flat, and shortness of breath. Her NT-proBNP on initial visit was 87 pg/mL (normal value <125 pg/mL).

## Differential Diagnosis

On the basis of the patient’s presenting symptoms and the relevant investigations, she received a diagnosis of acute pericarditis. Differential diagnoses included, but were not limited to, acute coronary syndrome, pulmonary embolism, gastroesophageal reflux disease, and costochondritis, all of which were systematically excluded.

## Investigations

**Figure 1:**
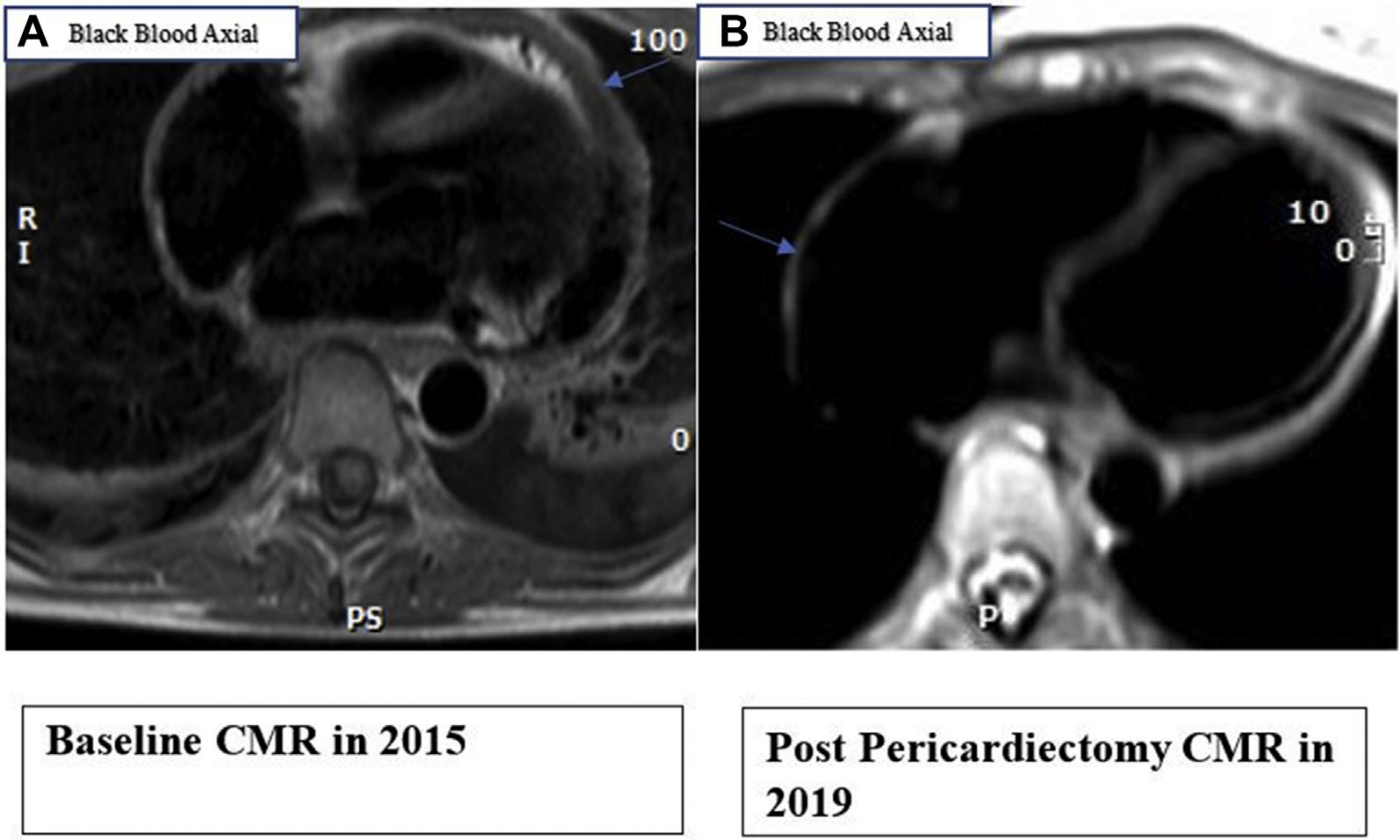
Cardiac MRI Black Blood Axial imaging **(A)** 2015 image showing increased pericardial thickening. **(B)** 2019 postpericardiectomy image; **blue arrow** demonstrates resection of pericardium.

**Figure 2:**
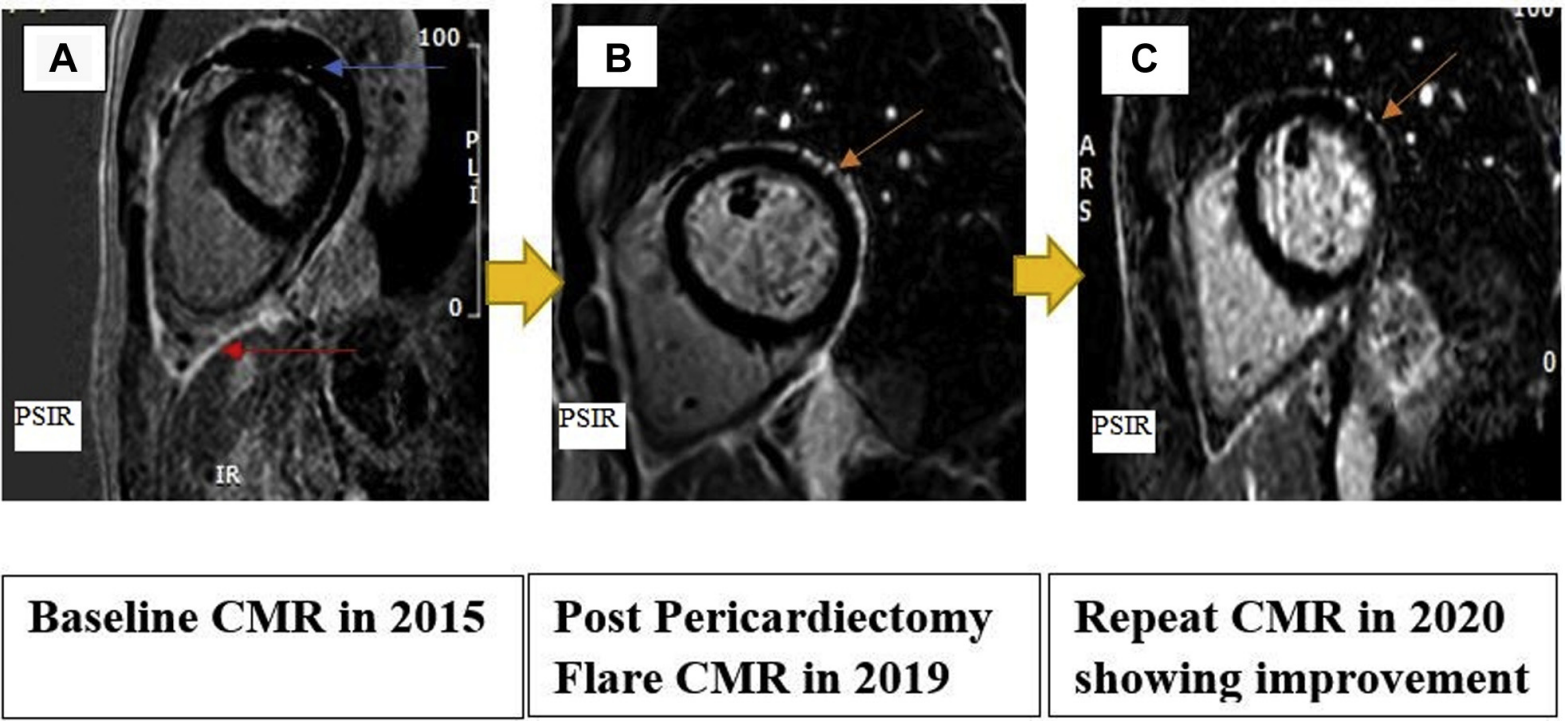
Cardiac Magnetic Resonance **(A)** 2015 phase-sensitive inversion recovery (PSIR) short axis image showing increased uptake on delayed enhancement (DHE) **(red arrow)** and loculated pericardial effusion **(blue arrow)** in 3D zoom PSIR. **(B)** 2019 image showing DHE in 3D zoom PSIR fat-suppressed sequence **(arrow)**. **(C)** 2020 image showing interval improvement in DHE in 3D zoom PSIR fat-suppressed sequence **(arrow)**. CMR = cardiac magnetic resonance.

As part of the clinical evaluation for recurrent pericarditis symptoms, cardiac magnetic resonance imaging (CMR) was performed 6 months later after the onset of her symptoms. Black blood imaging revealed a thickened pericardium (Figure 1). Delayed hyperenhancement imaging (DHE) with gadolinium-based contrast material demonstrated increase uptake in the pericardium, indicative of pericardial inflammation and a loculated pericardial effusion (Figure 2A). T2-weighted short T1 inversion recovery (STIR) sequences were of limited utility because of the presence of the pericardial effusion.

## Management

The patient was prescribed colchicine 0.6 mg twice a day, ibuprofen 400 mg three times daily, and prednisone 20 mg for 5 days. She also underwent emergent pericardiocentesis with drainage of approximately 0.5 L of serous fluid because of progression of her persistent pericardial effusion. She continued triple anti-inflammatory therapy with a slow taper of steroids.

Owing to the refractory and recurrent nature of her symptoms, despite optimal anti-inflammatory therapy she was referred to our complex pericardial disease center for further evaluation and optimization of her medical therapy. The inflammatory markers at the time of presentation included an erythrocyte sedimentation rate (ESR) that was elevated at 29 mm/h (normal range 0-15 mm/h) and a C-reactive protein (CRP) that was elevated at 2.8 mg/dL (normal range 0.0-1.0 mg/dL). Because of the prolonged steroid dependence, anakinra was started, and she experienced mild improvement while continuing the steroid taper. Because of the prolonged nature of her illness and the refractory recurrent symptoms of pericarditis, she underwent a radical pericardiectomy 2 years later. Surgical pathology revealed mild fibrosis with focal fibrinous pericarditis along with adhesion formation. This was a complete pericardiectomy, and the surgeon was confident that no residual pericardial tissue had been left adherent to the myocardium.

**Figure 3:**
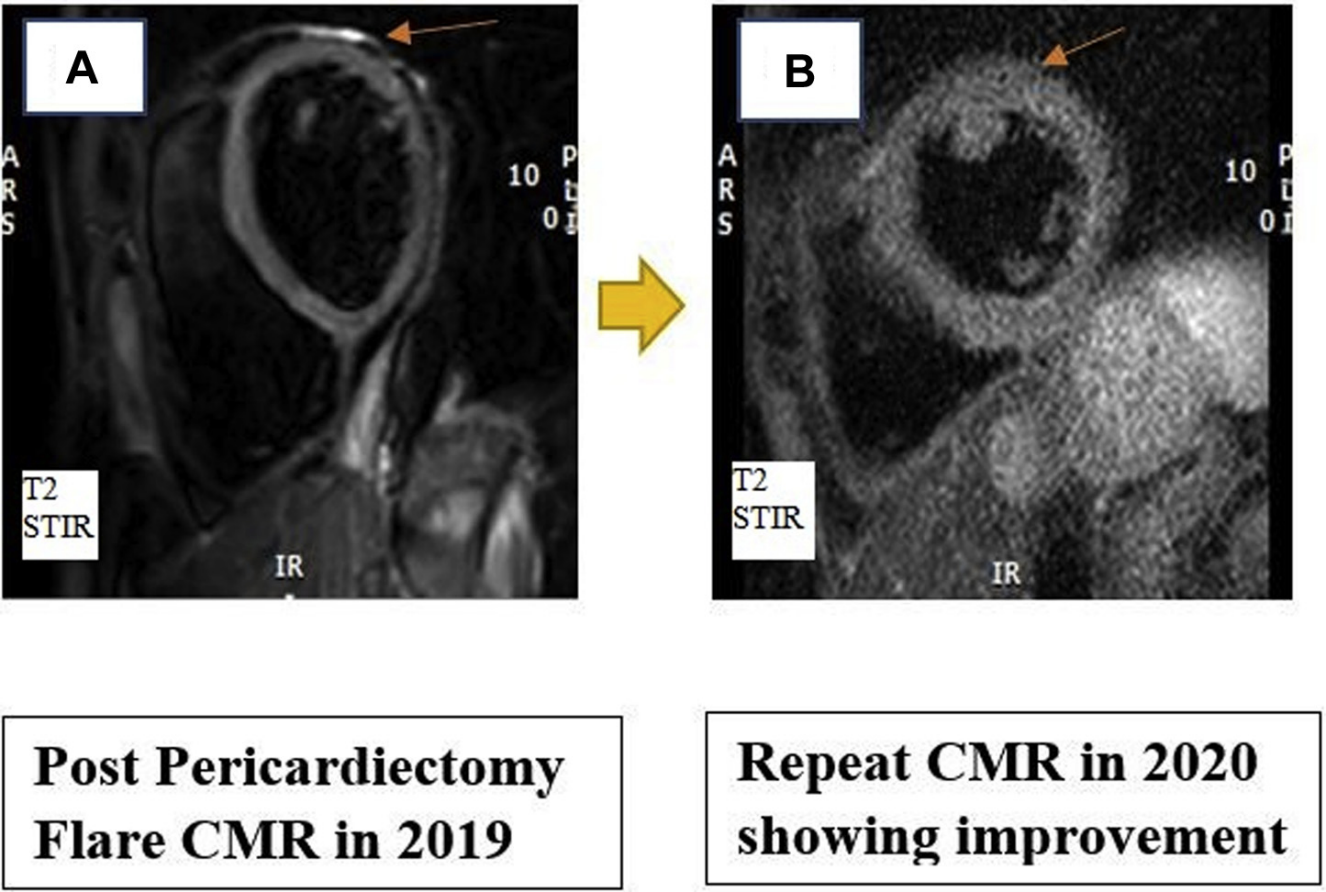
Cardiac Magnetic Resonance **(A)** 2019 image showing increased signal intensity on T2-weighted short T1 inversion recovery (STIR) imaging **(orange arrow)**. **(B)** 2020 image showing interval improvement in signal intensity in T2-weighted STIR sequence. CMR = cardiac magnetic resonance.

After the pericardiectomy, she reported worsening pleuritic chest pain, and the inflammatory markers were elevated, with an ESR of 15 mm/h and ultrasensitive CRP of 6.95 mg/dL while her anakinra was being tapered (Tables 1 and 2). A repeat CMR in 2019 with black blood axial imaging showed complete resection of the pericardium (Figure 1B), a gadolinium-based delayed hyperenhancement (DHE) in phase-sensitive inversion recovery (PSIR) sequence showed residual increased uptake pericardial area. This was also visualized on a fat-suppressed DHE sequence (Figure 2B). In addition, there was increased signal intensity on T2-weighted STIR and STIR zoom sequence, suggestive of edema (Figure 3A). Her autoimmune workup including serology was only weakly positive for antinuclear antibodies. There was no relevant family or personal history of connective tissue disease or vasculitis. She was not considered a candidate for repeat pericardiectomy.

**Table 1:** Trend of Inflammatory Markers

Markers	Latest Reference Range	May 2016	May 2016	September 2016	April 2017	October 2017	March 2018	February 2019	March 2019
ESR, mm/h	0-20		4	29 (H)	5	5	8		8
US-CRP, mg/dL	<3.1	<0.2	<0.2	34.7	0.3	0.3	<0.3	99.7	0.8

ESR = erythrocyte sedimentation rate; US-CRP = ultrasensitive C-reactive protein.

**Table 2:** Timeline of Events

Time	Event
July 2015	Mitral valve surgery for mitral valve prolapse
August 2015 to October 2015	First episode of pericarditis, started triple therapy including NSAIDs, colchicine, and steroids First CMR in October 2015
October 2015 to December 2017	Multiple recurrences, requiring a pericardiocentesis Owing to prolonged steroid dependence, started anakinra in September 2016, with mild improvement along with steroid taper Owing to the prolonged nature of her illness and persistent pain, patient underwent a successful radical pericardiectomy in December 2017
January 2018 to March 2019	Patient reported worsening pleuritic chest pain; elevation in inflammatory markers to ESR (15 mm/h) and US-CRP (6.95 mg/dL) while tapering anakinra CMR in 2019 demonstrated increased gadolinium uptake on DHE, and T2 STIR sequence showed increased signal intensity; anakinra was increased to once daily again, but patient continued to experience worsening symptoms; colchicine 0.6 mg orally twice daily was added to anakinra along with ibuprofen 400 mg three times daily as needed
April 2019 to September 2020	Repeat CMR in September 2020 demonstrated qualitative reduction in the intensity of gadolinium uptake on DHE imaging and normalization of signal intensity on T2 STIR imaging

CMR = cardiac magnetic resonance imaging; DHE = delayed hyperenhancement; ESR = erythrocyte sedimentation rate; STIR = short T1 inversion recovery; US-CRP = ultrasensitive C-reactive protein.

## Discussion

Our patient had chronic recurrent pericarditis due to post cardiac injury (post-pericardiotomy) from her previous mitral valve repair. Post-pericardiotomy syndrome (PPS) is an underappreciated condition with significant morbidity that occurs in predisposed patients after cardiothoracic surgery. Although the long-term prognosis of patients with PPS is generally favorable, flares of recurrent pericarditis develop in 10% to 15 % of patients, which can be debilitating (1). Most patients are responsive to anti-inflammatory therapy (NSAIDs, corticosteroids, or colchicine); however, a select subset of patients continue to experience recurrent flares of pericarditis despite optimal basic anti-inflammatory and steroid therapy. These patients may benefit from the earlier introduction of advanced immunosuppressive therapies such as interleukin-1 inhibitors such as anakinra or rilonacept or azathioprine, which should be initiated at a pericardial center of excellence that is experienced in the management of complex pericardial disease. It is reported that up to 5% of patients may require pericardiocentesis resulting from the development of a hemodynamically significant effusion, and a few undergo pericardiectomy (1,2).

### Clinical Signs and Symptoms

Patients with PPS present with symptoms of acute pleuritic chest pain and/or associated constitutional symptoms with shortness of breath. The clinical diagnosis is based on the presenting history, physical examination findings, and biochemical markers (ESR and US-CRP/CRP).

### Imaging

Multimodality cardiac imaging modalities, namely, transthoracic echocardiogram (TTE), computed tomography (CT) and/or CMR are used adjunctively to confirm the clinical diagnosis of pericarditis (3, 4, 5). TTE is primarily used as the initial diagnostic imaging modality of choice for the detection of biventricular function, assessment of segmental abnormality, and screen constrictive physiology and to evaluate for the presence of a pericardial effusion. TTE has no significant role in the confirmation of acute pericardial and/or myocardial inflammation, and it has limited utility in tissue characterization of the pericardium. In addition, owing to patient- and operator-related factors, suboptimal acoustic windows may result in limited imaging planes for accurate quantification of pericardial thickness. CT is predominantly used for the detection of pericardial calcification and/or thickening, in addition to further assessment of the size of a pericardial effusion (6). It is the modality of choice for assessing pericardial calcification and localizing the thickest segments of the pericardium. CMR provides morphologic and functional information on the heart; however. most importantly, it enables the clinician to characterize both myocardial and pericardial tissue. This is a key strength of CMR in comparison with other imaging modalities; CMR sequences are used to characterize the relaxation properties of the myocardium and pericardium both qualitatively and semiquantitatively, using native tissue relaxation properties or exogenous contrast-enhanced tissue relaxation times to assess for acute edema, inflammation, and/or fibrosis (7).

DHE imaging demonstrates increased vascularity in the presence of pre-existing pericardial inflammation (8). In our patient s/p pericardiectomy, circumferential enhancement may reflect epicardial/visceral pericardial inflammation or inflammation of epicardial fat in patients. STIR T2-weighted sequences detect acute inflammation by demonstrating increased signal intensity resulting from edema (9). DHE quantification also carries a prognostic role with as increased DHE is associated with a higher 6-month recurrence rate and a shorter time to relapse (10). The use of novel CMR-based imaging techniques such as T1 mapping may be of utility to detect active inflammation and/or gauge the adequacy of response to therapy. At present, these techniques are novel and are used primarily for research purposes; however, they are promising and require further validation in prospective outcome studies.

### Treatment

Surgical pericardiectomy is indicated in patients with symptomatic pericardial constriction, those with refractory recurrent pericarditis despite optimal medical therapy, and those with severe intolerance to medical therapy (11,12). The evidence for pericardiectomy in pericardial constriction is better established; however, the role for pericardiectomy in patients with refractory recurrent pericarditis is expanding. Gillaspie et al (13) demonstrated improved morbidity in patients with chronic relapsing pericarditis after pericardiectomy. In addition, Chiabrando et al (14) demonstrated that pericardiectomy may be used successfully as a therapeutic option for symptomatic relief in patients with relapsing pericarditis without evidence of constriction in treatment-refractory pericarditis. The conventional technique for radical pericardiectomy in the setting of recurrent pericarditis is to expose the pericardium via open pericardiectomy followed by comprehensive stripping/removal of the pericardium with decortication of the atria and ventricles (15).

In rare situations, patients may experience a relapse of pericarditis after pericardiectomy as in our patient. In a retrospective review of 184 patients by Khandaker et al (11), relapse was observed in 5 patients (8.6%) after pericardiectomy. Of interest, a small portion of the pericardium remains beneath the phrenic nerves in the vicinity of the diaphragm and posterior to the left atrium in oblique sinus that may serve as a potential source of pericardial inflammation (11). Postsurgical pericardial adhesions are also an unavoidable consequence of cardiothoracic surgeries and present with increased technical difficulties during reoperation (16). These pericardial adhesions are not completely removed during pericardiectomy and may serve as a potential nidus of future inflammation and recurrent pericarditis after pericardiectomy. Our case reflects this unusual presentation of ongoing chest pain despite surgical treatment.

## Follow-Up

The dose of anakinra was increased to once daily again, but the patient continued to experience worsening symptoms. Colchicine 0.6 mg orally twice daily was added to anakinra along with ibuprofen 400 mg three times daily as needed. Her symptoms were moderately controlled. A repeat cardiac MRI in 2020 demonstrated a qualitative reduction in the intensity of the gadolinium uptake on DHE imaging (Figure 2C) with normalization of signal intensity on T2 STIR imaging (Figure 3B).

## Conclusions

We present the case of a 40-year-old woman who experienced refractory recurrent pericarditis associated with PPS after mitral valve surgery, with a complex clinical course resulting ultimately in pericardiectomy. She continued to experience chest pain suggestive of a recurrence after pericardiectomy and was again given anti-inflammatory therapy for symptomatic relief.

## Funding Support and Author Disclosures

Dr Anthony is partially funded by the Braun Fund for Imaging Research, Cleveland Clinic. Dr Klein has received research funding from Kiniska Pharmaceuticals, Ltd, Swedish Orphan Biovitrum AB, and Pfizer, Inc; and is a member of the scientific advisory boards Kiniksa Pharmaceuticals, Ltd, Swedish Orphan Biovitrum AB, and Pfizer, Inc. All other authors have reported that they have no relationships relevant to the contents of this paper to disclose
